# Blueberry Husks and Probiotics Attenuate Colorectal Inflammation and Oncogenesis, and Liver Injuries in Rats Exposed to Cycling DSS-Treatment

**DOI:** 10.1371/journal.pone.0033510

**Published:** 2012-03-23

**Authors:** Åsa Håkansson, Camilla Bränning, Göran Molin, Diya Adawi, Marie-Louise Hagslätt, Bengt Jeppsson, Margareta Nyman, Siv Ahrné

**Affiliations:** 1 Food Hygiene, Division of Applied Nutrition and Food Chemistry, Lund University, Lund, Sweden; 2 Division of Applied Nutrition and Food Chemistry, Lund University, Lund, Sweden; 3 Department of Surgery, Lund University, Skåne University Hospital, Malmö, Sweden; Health Canada, Canada

## Abstract

Long-term colonic inflammation promotes carcinogenesis and histological abnormalities of the liver, and colorectal tumours frequently arise in a background of dysplasia, a precursor of adenomas. Altered colonic microbiota with an increased proportion of bacteria with pro-inflammatory characteristics, have been implicated in neoplastic progression. The composition of the microbiota can be modified by dietary components such as probiotics, polyphenols and dietary fibres. In the present study, the influence of probiotics in combination with blueberry husks on colorectal carcinogenesis and subsequent liver damage was evaluated.

Colorectal tumours were induced in rats by cyclic treatment with dextran sulphate sodium (DSS). Blueberry husks and a mixture of three probiotic strains (*Bifidobacterium infantis* DSM 15159, *Lactobacillus gasseri*, DSM 16737 and *Lactobacillus plantarum* DSM 15313) supplemented a basic diet fortified with oats. The condition of the rats was monitored using a disease activity index (DAI). A qualitative and quantitative histological judgement was performed on segments of distal colon and rectum and the caudate lobe of the liver. The formation of short-chain fatty acids, bacterial translocation, the inflammatory reaction and viable count of lactobacilli and *Enterobaceriaceae* were addressed.

Blueberry husks with or without probiotics significantly decreased DAI, and significantly reduced the number of colonic ulcers and dysplastic lesions. With a decreased proportion of blueberry husk in the diet, the probiotic supplement was needed to achieve a significant decrease in numbers of dysplastic lesions. Probiotics decreased faecal viable count of *Enterobacteriaceae* and increased that of lactobacilli. Blueberry husks with or without probiotics lowered the proportion of butyric acid in distal colon, and decreased the haptoglobin levels. Probiotics mitigated hepatic injuries by decreasing parenchymal infiltration and the incidence of stasis and translocation. The results demonstrate a dietary option for use of blueberry husks and probiotics to delay colonic carcinogenesis and hepatic injuries in the rat model.

## Introduction

Cancers may arise from sites of infection, chronic irritation and inflammation [1] and the degree and extent of inflammation during, for example, ulcerative colitis (UC), is a critical component of tumour development and progression [2]. UC-associated colorectal tumours frequently arise in a background of dysplasia and differ in pathogenesis and molecular features from sporadic colorectal cancer [3]. The presence of dysplasia-associated lesions is highly indicative for underlying or associated cancer [4]. Histological abnormalities of the liver of patients with chronic UC have been observed [5], and steatosis and primary sclerosing cholangitis are the most common lesions [6].

The cause of gastrointestinal tumours is implicating chronic inflammation in response to an adverse bacterial flora as a promotion of neoplastic progression, and the intestinal environment is considered important in both colorectal cancer development and modulation of mucosal immunity [7]. During inflammation in UC-patients, different members of the *Enterobacteriaceae* family and different *Clostridium* species have been found to increase in accordance with a decrease in bifidobacteria and lactobacilli [8], [9]. This change in the composition of the microbiota, leading to an imbalance between potentially beneficial and adverse bacteria, can contribute to the pathogenesis. Lipopolysaccharides (LPS) associated to the cell wall of Gram-negative bacteria, are highly inflammatory compounds. LPS are associated with disturbed mucosal integrity, and bacterial translocation from the GI- tract [10]. Translocated LPS can cause extensive damage to a variety of organs, including the liver [11].

Dietary-induced changes in the different populations of the intestinal microbiota can be achieved by use of dietary fibres and/or probiotics. Probiotics may affect the intestinal flora but may also modulate immunological functions and affect intestinal permeability which will have effects on bacterial translocation [12] and liver health. Microbial degradation in the hindgut of dietary carbohydrates escaping digestion, results in production of short-chain fatty acids (SCFAs), mainly acetic acid, propionic acid and butyric acid [13]. Butyrate is considered to be the preferred source of energy for colonocytes but to some extent also propionic acid can be utilized [14]. Fermentation of dietary oat-fibres results in elevated amounts of butyric acid [15], which has been suggested to mitigate colorectal cancer development [16].

Other dietary components, such as various phenolic compounds, may modulate the composition of the intestinal microflora [17]. Blueberries are rich in a variety of phenolics, which have been shown to inhibit colon cancer and cell proliferation, and induce apoptosis in vitro [18].

DSS is a non-genotoxic, sulphated, polysaccharide that nevertheless can induce experimental chronic colitis and colitis-associated neoplasia in animals. The histopathological changes show reminiscence of human UC [19]. During long-term DSS exposure, dysplasia and/or cancer occurs as dysplasia-associated lesions, which has similarities to the development of dysplasia and cancer development in humans with colitis [19]. However, the effect on the liver of long-term DSS-induced colitis is mainly unknown. The aim of the present study has been to evaluate the potential of blueberry husks and a probiotic mixture to attenuate inflammatory injuries in colon and liver and to mitigate colonic dysplasia development. It was supported by evidence that the faecal flora was influenced and linked to changed profiles of SCFA production, the hepatic damage was affected and carcinogenic progression was delayed.

## Methods

### Ethics Statement

The Ethics Committee for Animal Studies at Lund University approved the animal experiment (permit number and approval-ID: M25-06).

### Animals and experimental design

Female Sprague-Dawley rats (n = 48), purchased from Scanbur (Sollentuna, Sweden), were housed four per cage at room temperature of 22°C with 12 h light/dark cycles and given free access to water, while feed intake was restricted to 92 g (dwb, dry weight basis) per cage and day. Animals were randomly divided into six groups with eight animals in each group, and given different diets according to Table 1. In the diets, oat bran and blueberry husks were the sources of dietary fibres (Table 1). The blueberry husks were derived from pressed wild low-bush blueberry of *Vaccinium myrtillus* L, and were freeze-dried before inclusion (Probi AB, Lund, Sweden), and the oat bran (*Avena sativa* L. cv. Sang) was supplied by Lantmännen (Järna, Sweden). A mixture of freeze-dried *Bifidobacterium infantis* DSM 15159 ( = CURE21; dose, 2⋅10^9^ CFU/d), *Lactobacillus gasseri* DSM 16737 ( = VPG44; dose, 1⋅10^9^ CFU/d) and *Lactobacillus plantarum* DSM 15313 ( = HEAL19; dose, 3⋅10^9^ CFU/d) was added to the diet for three of the groups (Table 1). Oat bran and blueberry husks were included at a level of 50 g dietary fibre/kg in the diets (dwb) for groups C (oat bran), 2B (blueberry husks) and 2BP (blueberry husks and probiotics) (Table 1). The soluble and insoluble dietary fibres in the raw materials were determined by a gravimetric method [20]. The composition of the fibre residues was analysed by gas-liquid chromatography (GLC) for the neutral sugars as their alditol acetates and spectrophotometrically for the uronic acids [21]. In the blueberry diets marked B and BP (P = probiotics; Table 1), half of the amount of dietary fibre (25 g dietary fibre/kg) comprised oat bran and the other half comprised blueberry husks. The amount of fibre was chosen to approximate a moderate fibre intake, corresponding to an equivalent dose of around 30 g fibre/d in humans. The dry matter content was adjusted with wheat starch, and the dietary fibre content was 17.1 g/100 g (dwb) in oat bran and 40.8 g/100 g (dwb) in blueberry husks (Table 1). Of the dietary fibre content in oat bran, 1.5 g/100 g (dwb) was Klason lignin, i.e. components not soluble in 12 M H_2_SO_4_, whereas the amount of Klason lignin in blueberry husk was 14.1 g/100 g (dwb). The non-starch polysaccharides consisted mainly of glucose (61%), xylose (19%), and arabinose (12%) in oat bran and glucose (39%), uronic acids (25%) and xylose (20%) in blueberry husks. After 7 days of adaptation to the diets, an experimental period of 6 months followed, when feed residues were collected daily.

**Table 1:** Diet composition.

Component	C	P	2B	2BP	B	BP
Oat bran	291^1^	291^1^	-	-	145^2^	145^2^
Blueberry husks (B)	-	-	122^1^	122^1^	61^2^	61^2^
Basal mixture^3^	369.2	369.2	369.2	369.2	369.2	369.2
Wheat starch^4^	380	380	549	549	465	465
Probiotics (P)^5^	-	+	-	+	-	+

Composition of diets (g/kg dwb) given to rats in the following groups: Group 1, active control (C); Group 2, probiotics (P); Group 3, blueberry husks (2B); Group 4, blueberry husks and probiotics (2BP), reduced amount of blueberry husks (B), and reduced amount of blueberry husks and probiotics (BP).

1 Corresponding to 50 g dietary fibre/kg diet (dwb).

2 Corresponding to 25 g dietary fibre/kg diet (dwb).

3 Containing (g/kg dwb) 160 casein, 1.2 DL-methionine, 50 maize oil, 48 mineral mixture (Containing (g kg^−1^) 0.55 CuSO_4_⋅H_2_O, 2.0 ZnSO_4_⋅7H_2_O, 498 KH_2_PO_4_, 258 NaH_2_PO_4_⋅2H_2_O, 487 CaCO_3_, 0.1 KI, 86 MgSO_4_, 12 FeSO_4_⋅7H_2_O, 5 MnSO_4_⋅H_2_O, 0.03 CoCl⋅6H_2_O, 153 NaCl, 0.02 CrCl_3_⋅6H_2_O, 0.02 Na_2_Se), 8 vitamin mixture (Containing (g kg^−1^) 0.62 menadion, 2.5 thiamin hydrochloride, 2.5 riboflavin, 1.25 pyridoxin hydrochloride, 6.25 calcium pantothenate, 6.25 nicotinic acid, 0.25 folic acid, 12.5 inositol, 1.25 p-aminobenzoic acid, 0.05 biotin, 0.00375 cyanocobalamin, 0.187 retinol palmitate, 0.00613 calciferol, 25 d-α- tocopheryl acetate, 941.25 maize starch), 2 choline chloride, 100 sucrose.

4 Wheat starch (Cerestar, Krefeld, Germany).

5 +Freeze-dried *Bifidobacterium infantis* DSM 15159 ( = CURE21; dose, 2⋅10^9^ CFU/d), *Lactobacillus gasseri* DSM 16737 ( = VPG44; dose, 1⋅10^9^ CFU/d) and *Lactobacillus plantarum* DSM 15313 ( = HEAL19; dose, 3⋅10^9^ CFU/d) were added to the diet.

All groups were administered 4% (w/v) DSS (MW = 36,000–50,000; ICN Biomedicals Inc., Aurora, OH) dissolved in drinking water for 7 days, followed by 10 days of tap water, and this cycle was then repeated 11 times. The DSS solution was changed daily. Rats were weighed before and after the adaptation period, as well as daily during the DSS consumption. Body weight change during the experimental period was calculated as gram per animal or as body weight change per kilo gram feed consumed and animal. An attempt was made to quantify the amount of drinking water and DSS load ingested by the rats. Drinking volumes were recorded every 24 h for each cage (four animals) and the DSS load per animal was calculated over the experimental period as:
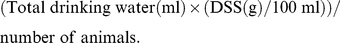



### Sampling

Blood samples for analysis of haptoglobin were taken from the saphenous vein at the beginning of the study, and during cycle 1, 5 and 10. During each DSS cycle samples were taken on the seventh day of DSS administration and on the tenth day of the subsequent water period. At the same time faecal samples were collected for viable count.

The animals were anaesthetised with Hypnorm (Division of Janssen-Cilag Ltd., Janssen Pharmaceutica, Beerse, Belgium), Dormicum (F. Hoffman-La Roche AG, Basel, Switzerland) and water (1∶1∶2) at a dose of 0.15 ml/100 g of body weight by a subcutaneous injection. Arterial blood was collected for analysis of SCFAs and liver specimens were obtained for bacterial translocation and liver histology. The entire colorectum from the colocaecal junction to the anal verge was excised and the luminal content of caecum and colon was gently removed for analysis of SCFAs and pH was measured in caecal content before storage at −40°C. The large bowels were macroscopically examined for gross lesions all of which were recorded, and then the colons were cut and fixed in 10% buffered formalin, for 24 h.

### Clinical scoring of colitis

Disease severity was analysed in terms of disease activity index (DAI), calculated on the basis of weight loss, stool consistency, and bleeding. The scoring system has been validated [22] and shown to correlate histologically with pathological findings [23]. The DAI was assessed daily during DSS administration from day 0 to day 7 and scored on a scale of 0–4 for each clinical parameter and then averaged for each animal. Weight-loss, stool and bleeding scores were defined by modified scoring limits [24].

### Haptoglobin

The concentration of serum haptoglobin was analysed using a manual microplate (96 microwell plates, Nunc™, Roskilde, Denmark) method. In this assay, serum was incubated with haemoglobin (Hb) (0.12 mg/ml bovine haemoglobin (Sigma Aldrich, St Louis, USA) in 0.15 M NaCl (Merck Schuchardt, Hohenbrunn, Germany)) leading to preserved peroxidase activity of the complex. The preserved activity, measured by the addition of a peroxidase substrate (chromogenic solution; 0.5 M citrate buffer pH 3.8 (0.5 M sodium citrate dihydrate (J.T Baker B.V., Deventer, Holland), 0.5 M citric acid-1-hydrate (Merck)), 1% Tween 20 (Merck), 20 mM phenol (International Biotechnologies Inc., Eastman Kodak Co. Rochester, NY), 0.39 mM dithioerythritol (Sigma), 1.6 mM 4-aminoantipyrine (Sigma), 1 mM 8-anilino-1-naphthalene sulphonic acid (Sigma) and 1 µl 30% H_2_O_2_/0.7 ml solution (Merck), is directly proportional to the amount of haptoglobin in the samples. Absorbance was measured at 600 nm (SpectraMax® M2 Multi-detection Microplate Reader, Molecular Devices, Sunnyvale, California) and the results were compared with a haptoglobin standard (2 mg/ml) (Tridelta Development Ltd, Maynouth County Kildare, Ireland).

### Histological evaluation

Specimens from the distal part of colon and from the liver were evaluated by light microscopy. Macroscopic abnormalities through the entire length of colon, and microscopic alterations, were evaluated by an experienced surgeon and pathologist respectively. The biopsies of the distal colon, taken at selected sampling sites (polyps or dysplastic lesions and surrounding mucosa), and the left lobe of the liver, were each fixed in neutral buffered formalin, followed by standard procedure for paraffin embedding. Serial sections were cut for each organ and stained with haematoxylin-eosin staining. The degree of dysplasia in the colon was scored from normal to mucosa with mild dysplasia (with distorted crypts of increased length and orientation), and mucosa with severe dysplasia (with severe crypt distortion, atypic epithelial cells, reduction or loss of goblet cells, hyperchromatic cell nuclei and increased numbers of cell mitoses). Liver specimens were evaluated for the degree of steatosis according to Brunt *et al.*, [25], where steatosis was scored as absent ( = 0), mild when present in <1/3 of the hepatocytes ( = 1), moderate when present in 1/3–2/3 of the hepatocytes ( = 2), and severe when present in >2/3 of the hepatocytes ( = 3). The presence and location of infiltrating inflammatory cells and liver injury was also recorded. The degree of infiltrating inflammatory cells in steatotic and non-steatotic areas, vascular stasis and loss of liver parenchyma in either zonal or nonzonal distribution were measured using a semi-quantitative graded scale of 0 (absent), 1 (mild), 2 (moderate), and 3 (extensive) [26], to enable statistical evaluation.

### Bacterial translocation

Samples from the caudate lobe of the liver were removed aseptically and frozen immediately at −70°C until determination. For analysis, the samples were thawed, placed in an ultrasonic bath (Millipore, Sundbyberg, Sweden) for 5 min and swirled for 2 min on a Chiltern. Viable counts were obtained from Violet-red bile glucose (VRBG) agar (Oxoid) that was incubated aerobically at 37°C for 24 h (*Enterobacteriaceae* count), brain heart infusion (BHI) agar (Difco, Detroit, MI) that was incubated aerobically and under anaerobic conditions, as described above, at 37°C for 72 h (total aerobic and anaerobic counts, respectively), and from Rogosa agar (Oxoid), incubated anaerobically at 37°C for 72 h (lactobacilli count). Results were expressed as incidences of positive cultures/group.

Colonies were randomly picked from the plates with positive cultures and identified by sequencing the 16 S ribosomal RNA gene. The partial 16 S rRNA gene sequences were searched against GenBank (National Centre for Biotechnology Information, Bethesda, MD) using the Basic Local Alignment Search Tool (BLAST) accessible from the homepage at the National Centre for Biotechnology Information (NCBI; http://www.ncbi.nlm.nih.gov/). The compared sequence lengths were between 200–900 base pairs.

### Viable count of *Enterobacteriaceae* and lactobacilli in faeces

Faecal samples were thawed and homogenised in freezing medium, diluted (sodium chloride (Merck), 8.5 g/l; Bacteriological peptone (Oxoid, Unipath LTD Basingstoke, Hampshire, England), 1 g/l; Tween 80 (Merck), 1 g/l; L-Cystine hydrochloride monohydrate (Merck), 0.2 g/l) and plated on Rogosa agar for lactobacilli count (Oxoid; incubated anaerobically [Gas Pack System, Gas Pack; Becton Dickenson Microbiology Systems, Cockeynsville, MD] at 37°C for 72 h) and violet red bile-glucose agar (VRBG) for *Enterobacteriaceae* count (Oxoid; incubated aerobically at 37°C for 24 h). The number of colonies formed on each plate was counted and corrected for the weight of the original faecal sample, and expressed as CFU/g faeces.

Colonies were randomly picked from countable Rogosa agar plates in order to get an idea of the identity of the dominant faecal lactobacilli flora. Colonies from four different time-points were collected (before adaptation period, day 7 of DSS cycle 1, day 7 of DSS cycle 10, during surgery) and a total of 56 isolates were identified through RAPD band pattern comparison and nucleotide sequencing. Obtained sequences were at least 700 base-pair long and the results showed no less than 99% sequence similarity to their nearest database entries.

### Randomly Amplified Polymorphic DNA (RAPD) analysis

As template for the polymerase chain reaction, crude cell extract was prepared [27] and one microlitre of PCR template was used in the polymerase chain reaction (PCR) [27]. Agarose gel (Type III, High EEO, Sigma) electrophoresis was run, and the gels were stained with ethidium bromide and photographed under UV illumination. RAPD band comparison of isolates taken from the feed was used for identification of *Lactobacillus plantarum* HEAL19.

### 16 S rDNA sequencing

For sequencing, primers ENV1 (5′-AGA GTT TGA TII TGG CTC AG-3′, *Escherichia coli* numbering 8–27) and ENV2 (5′-CGG ITA CCT TGT TAC GAC TT-3′, *E. coli* numbering 1511–1492) [28] were used for amplification of the 16 S rRNA genes. The PCR reaction mixture contained 0.2 µM of both primers, 5 µl of template DNA, 5 µl of 10× PCR reaction buffer with 1.5 mM MgCl_2_ (Roche Diagnostics GmbH, Mannheim, Germany), 200 µM of each deoxyribonucleotide triphosphate, and 2.5 U of Taq DNA polymerase (Roche Diagnostics, Mannheim, Germany). Water was added to a final volume of 50 µl. PCR was performed in a PCR Mastercycle 5333 (Eppendorf) with the following profile: 1 cycle at 94°C for 3 min, followed by 30 cycles of 96°C for 15 s, 50°C for 30 s, and 72°C for 90 s, with an additional extension at 72°C for 10 min. The amplification products (5 µl) were checked by running the products on 1.5% (wt./vol.) agarose gel in 1× TBE buffer (89 mM Tris, 89 mM boric acid, 2.5 mM EDTA, pH 8.3), after ethidium bromide staining. Amplicons were sent to MWG (Biotech, Ebersberg, Germany) for single strand sequencing. 16 S rDNA sequences (mostly around 500 bp) were searched against Genbank (blastn) option at the homepage of the National Centre for Biotechnology (http://www.ncbi.nlm.nih.gov/BLAST/) [29] or aligned to 16 S rDNA encoding sequences downloaded from the Ribosomal Data Base (RDP-II) [30] for an approximate phylogenetic affiliation.

### Short-chain fatty acids

The short-chain fatty acids (SCFAs; acetic, propionic, isobutyric, butyric, isovaleric and valeric acids) were analysed in serum using GLC [31] with small modifications. Water and 2-ethylbutyric acid (internal standard) were added to the serum samples and the SCFAs were protonised with hydrochloric acid. The caecal and colonic amounts of SCFAs (acetic, propionic, isobutyric, butyric, isovaleric, valeric, caproic and heptanoic acids) were analysed by a GLC method [32] with minor modifications. Water mixed with hydrochloric acid and 2-ethylbutyric acid was added to the faecal samples before homogenisation of the suspension.

### Statistics

**Figure 1:**
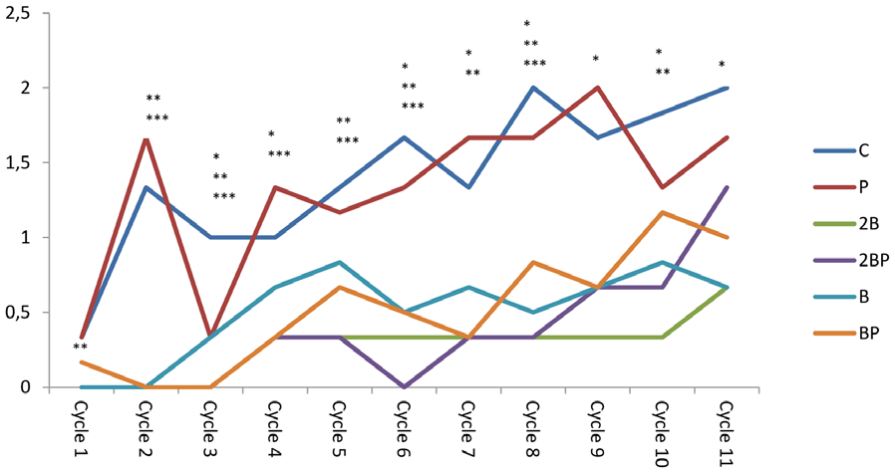
Disease activity index. Disease activity index (DAI) during the 11 cycles of DSS administration. DAI scores are expressed as medians (25 and 75 percentiles). Significant differences are expressed versus the C group. Cycle 1: **P<0.01 for groups 2B, 2BP and B; Cycle 2: **P<0.01 for groups B and BP, *** P<0.001 for groups 2B and 2BP groups; Cycle 3: *P<0.05 for group P, **P<0.001 for group B group, ***P<0.001 for groups 2B, 2BP and BP; Cycle 4: *P<0.05 for group BP, ***P<0.001 for groups 2B and 2BP; Cycle 5: **P<0.01 for group B group, ***P<0.001 for groups 2B, 2BP, and BP; Cycle 6: *P<0.05 for group B, **P<0.01 for group BP,***P<0.001 for groups 2B and BP; Cycle 7: *P<0.05 for group B, **P<0.01 for groups 2B and B, ***P<0.001 for groups 2BP and BP; Cycle 8: *P<0.05 for group BP, **P<0.01 for groups 2B and B, ***P<0.001 for group 2BP; Cycle 9: *P<0.05 for groups 2B, 2BP, B, BP; Cycle 10, *P<0.05 for group B, **P<0.01 for groups 2B and 2BP; Cycle 11: *P<0.05 for groups 2B, 2BP, B, BP.

**Figure 2:**
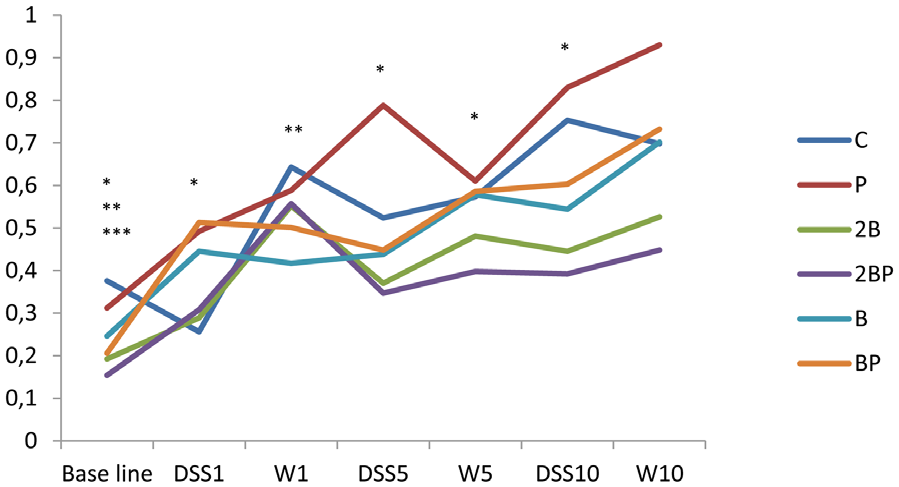
Concentrations of haptoglobin. Haptoglobin concentrations (mg/ml) in blood during the 11 cycles of DSS administration. Concentrations are expressed as medians (25 and 75 percentiles). Significant differences are expressed versus the C group. Base line: *P<0.05 for group B, **P≤0.01 for group 2B group, ***P<0.001 for group 2BP. DSS cycle 1: *P<0.05 for group BP. DSS cycle 5: *P<0.05 for group 2B. DSS cycle 10: *P<0.05 for group 2B. Pure water cycle (W1): **P<0.01 for group B; Water cycle 5 (W5) *P<0.05 for group 2B group.

DAI scores, haptoglobin, number of dysplastic lesions, number of ulcers, scoring used for histopathologic evaluation of liver samples, lactobacilli and *Enterobacteriaceae* counts (Fig. 1, 2, 5, 6, 11, 12 and Table 2) were presented as medians with 25 and 75 percentiles. The statistics were conducted in SigmaStat® version 3.0 (SPSS Inc., Chicago, Ill., USA). Differences between all groups were evaluated by Kruskal-Wallis test one way ANOVA on ranks followed by all pairwise multiple comparison procedures (Student-Newman-Keuls method), if appropriate. The differences between treatment groups were assessed by a Mann-Whitney rank sum test. Incidence of steatosis, cellinfiltration, stasis, loss of parenchyma and translocation to the liver (Table 3, 4), as well as statistical comparison of the total received score compared to maximum score (Table 2) were calculated in QuickStat version 2.6 and evaluated by the Fisher exact test.

**Table 2:** Evaluation of liver injury (Scoring values).

Scoring values					
Groups	Steatosis	Cellinfiltration^1^	Cellinfiltration^2^	Stasis	Loss of parenchyma
C	10/24 (41.7%)	2/24 (8.3%)	16/24 (66.7%)	8/24 (38.1%)	6/18 (33.3%)
P	20,5/24*# (85.4%)	4/24 (16.7%)	16/24 (66.7%)	8/15 (53.3%)	4/24 (16.7%)
2B	5/24 (20.8%)	0/24	15/24 (62.5%)	12/24 (50.0%)	10/21 (47.6%)
2BP	15/24# (62.5%)	0/24	10/24* (41.7%)	8/24 (33.3%)	3/24 (12.5%)
B	6/15 (40.0%)	1/15 (6.7%)	6/15* (40.0%)	0/12	0/12
BP	16.5/21*# (78.6%)	1/18 (5.6%)	4/18* (22.2%)	0/18	0/18

Liver specimens were histologically evaluated for the degree of steatosis, infiltrating inflammatory cells in steatotic and non-steatotic areas, vascular stasis and loss of liver parenchyma. The status of the livers of different groups is expressed as scoring values (degree of scoring and received value/maximum value). Between brackets are the percentages of the values.

1 Cellinfiltration around CV within steatotic areas.

2 Cellinfiltration elsewhere in the parenchyma.

Degree of scoring: * denotes P<0.05 compared with the C group.

Statistical comparison of the total received score compared to maximum score: # denotes P<0.01 compared to rats fed diets without bacteria.

**Table 3:** Evaluation of liver injury (Incidence).

Incidence					
Groups	Steatosis	Cellinfiltration^1^	Cellinfiltration^2^	Stasis	Loss of parenchyma
C	8/8	1/8	8/8	4/7	3/6
P	8/8	2/8	8/8	4/5	2/8
2B	4/8 *	0/8	8/8	6/8	5/7
2BP	8/8	0/8	7/8	4/8	2/8
B	3/5	1/5	5/5	0/4	0/4
BP	7/7	1/6	4/6	0/6 *	0/6

Liver specimens were histologically evaluated for the incidence of steatosis, infiltrating inflammatory cells in steatotic and non-steatotic areas, vascular stasis and loss of liver parenchyma. The status of the livers of different groups is expressed as incidence of phenomena.

1 Cellinfiltration around CV within steatotic areas.

2 Cellinfiltration elsewhere in the parenchyma.

Incidence: * denotes P<0.05 compared with the C group.

**Table 4:** Incidence of translocations and identified isolates from the livers.

Groups	Translocation	Identity of isolate	Similarity to strain of known identity (%)
C	3/8; 2/8; 4/8	*L. animalis*	99 (2 isolates)
		*L. apodemi*	99
		*K. rhizophila*	99
		*L. frumenti (L. antri)*	96
		*L. antri*	99, 93
		*L. gasseri*	100
		*M. luteus*	100
		*C. ramosum*	100
		*S. warneri*	100 (2 isolates)
P	0/8; 2/8; 1/8	*C. ramosum*	100
		*L. xyli* subsp. *cynodontis*	99
2B	1/8; 1/8; 3/8	*L. animalis (L. apodemi)*	99
		*L. apodemi*	99 (2 isolates), 100
		*E. casseliflavus*	100
		*C. perfringens*	99
		*L. oris (L. frumenti, L. antri)*	97
		*L. gasseri*	100
		*K. rhizophila*	99
		*P. lautus*	99
2BP	0/8; 3/8; 3/8	*K. rosea (K. rhizophila)*	99
		*K. rosea*	99
		*M. luteus*	100
		*S. warneri*	100
		*L. animalis*	98
B	0/6; 1/6; 4/6	*C. lipophiloflavum*	99
		*C. subterminale*	98
		*C. perfringens*	100
		*K. rhizophila*	99
		*B. siralis*	99
BP	0/8; 0/8; 0/8 *	-	-

Thirty-four isolates were subjected to 16 S rDNA sequencing and the similarity levels for each isolate are shown in the table. Translocation is mentioned as incidence of phenomena/total number of animals. Isolates were received from Rogosa agar/Brain Heart Infusion agar (incubated anaerobically)/Brain Heart Infusion agar (incubated aerobically). Between brackets are species with the same sequence similarity as the before mentioned.

Incidence: * denotes P<0.05 compared with the C group.

*Lactobacillus animalis* (*L. animalis*), *Lactobacillus apodemi* (*L. apodemi*), *Kocuria rhizophila* (*K. rhizophila*), *Lactobacillis frumenti* (*L. frumenti*), *Lactobacillus antri* (*L. antri*), *Lactobacillus gasseri* (*L. gasseri*), *Micrococcus luteus* (*M. luteus*), *Clostridium ramosum* (*C. ramosum*), *Staphylococcus warneri* (*S. warneri*), *Leifsonia xyli* subsp. *cynodontis* (*L. xyli subsp. cynodontis*), *Enterococcus casseliflavus* (*E. casseliflavus*), *Clostridium perfringens* (*C. perfringens*), *Paenibacillus lautus* (*P. lautus*), *Kocuria rosea* (*K. rosea*), *Corynebacterium lipophiloflavum* (*C. lipophiloflavum*), *Clostridium subterminale* (*C. subterminale*), *Bacillus siralis* (*B. siralis*).

One-way ANOVA was used for individual means to assess the effect of dietary fibre or probiotics by using Tukey' procedure (SCFAs in colon content and blood samples). When error variance was found to be heterogeneous, data was transformed by BoxCox-transformation before ANOVA. Values are presented as means and differences resulting in P≤0.05 were considered significant.

## Results

### Body weight change

Body weight differed between groups at the start. Compared with the C group (189.5 g/animal (181.5–191.0), the animals of the 2BP group and BP group had a slightly higher body weight, 197.5 g/animal (194.0–202.5) (P = 0.001) and 200.0 g/animal (190.0–206.5) (P = 0.038)), respectively.

During the study, the feed intake was similar for all groups, and all animals gained weight with time, and at the end there were no significant differences between the groups, irrespective of the approach of calculation, i.e. the body weight change (g/animal) was for the C group 173.5 g (167.0–217.5), P group 194.5 g (158.0–217.5), 2B group 173.5 g (149.0–211.5), 2BP group 181.5 g (173.5–204.0), B group 153.0 g (138.0–211.0), BP group 163.0 g (146.5–196.5), and body weight change in relation to amount of consumed food (g/kg feed/animal) was for the C group 56.0 g (53.9–70.2), P group 62.7 g (51.0–70.2), 2B group 54.2 g (46.6–66.1), 2BP group 55.0 g (52.6–61.8), B group (47.8 g (43.1–65.9), BP group 54.3 g (48.8–65.5). Mean total consumption of DSS (in water) was 21 g/rat during the experimental period with no differences between the groups.

### Disease severity scoring

After the first DSS cycle the DAI score was significantly lower in groups 2B (P = 0.007), 2BP (P = 0.002) and B (P = 0.005) than in group C (Fig. 1). Groups supplemented with blueberry husks continued to have lower DAI than the C group through the different cycles. The P group only reached significant difference in the 3^rd^ cycle (P = 0.05). The signs of colitis gradually disappeared during the first periods with pure water, but DAI gradually increased over time and did not revert between the cycles of DSS administration (Fig. 1). At the 11^th^ cycle, a significantly lower DAI was found compared with group C for groups 2B (P = 0.015), 2BP (P = 0.021), B (P = 0.043) and BP (P = 0.050) (Fig. 1). All animals exhibited body weight loss and most of them showed more or less rectal bleeding and loose stool, and DAI increased significantly between the first and eleventh cycle of DSS administration for all groups (P<0.01). The mortality rate was 0%.

### Haptoglobin

The haptoglobin level in blood at base line was significantly lower in groups B (P = 0.043), 2B (P = 0.001) and 2BP (P<0.001) compared with that of group C (Fig. 2). Over the experimental period there is a general pattern that group P has higher haptoglobin values than group C, while all groups supplemented with blueberry husks have lower values (groups 2B, 2BP, B and BP; Fig. 2). A significant increase in haptoglobin levels from the base line to the end of the water period of cycle 10 was shown in all groups: C group (P = 0.01), P group (P<0.001), 2B group (P<0.001), 2BP group (P<0.001), B group (P = 0.002), BP group (P = 0.043) (Fig. 2).

### Histological and macroscopic alterations of colon

Macroscopic examination of colon from each animal revealed visible thickening of the colon walls in all groups. Invaginations as a cause of polyps and dilated descending colon were occasionally seen in animals of groups C, B and BP. No polyps were found in the groups 2B and 2BP.

**Figure 3:**
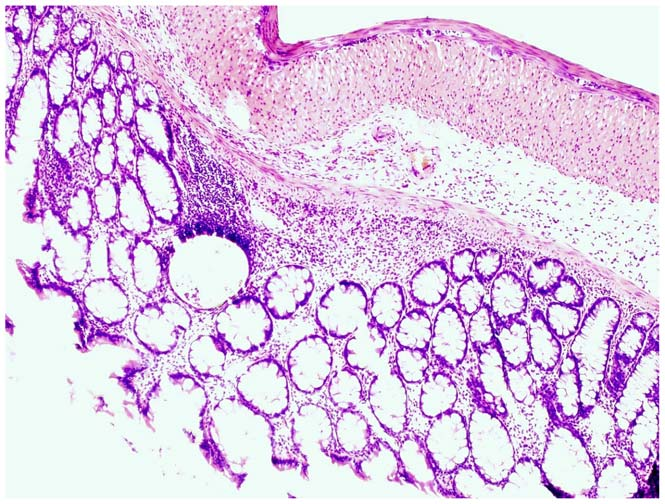
Flat dysplasia. Colonic mucosa showing flat dysplasia. The sample was taken from an animal in group C.

**Figure 4:**
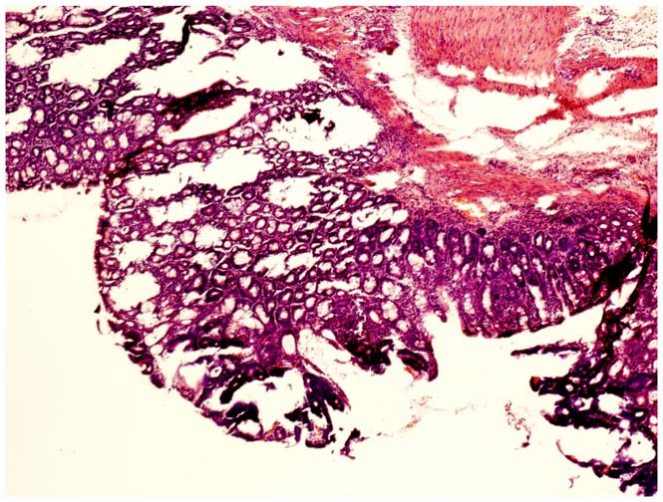
Dysplasia-associated lesion. Colonic mucosa showing a dysplastic lesion in association with inflammation. The sample was taken from an animal in group C.

Examination in the microscope shows that group C had colonic inflammation, mostly confined to the mucosa and submucosa, with loss of surface epithelium, inflammatory cell infiltrations, loss of goblet cells, crypt distortion and abscesses, mucosal ulceration and erosion, and accompanying submucosal edema (Fig. 3, 4). The diseased condition was found throughout the colon but was particularly prominent on the left side and in the transverse colon. Regenerative and hyperplastic epithelium, which morphologically mostly resembled low-grade dysplasia with some sections of high grade dysplasia and polyps diagnosed as adenocarcinomas, was observed (Fig. 3, 4). The overall histological changes in group P were similar to those of group C, while mucosal injuries were less severe in groups BP, B, 2BP and 2B. In groups 2B and 2BP, no sections of high grade dysplasia and adenomatous polyps were found. None of the animals in groups 2B, 2BP, B and BP showed gross mucosal ulceration. The bleeding per rectum was caused by small and focal erosions.

**Figure 5:**
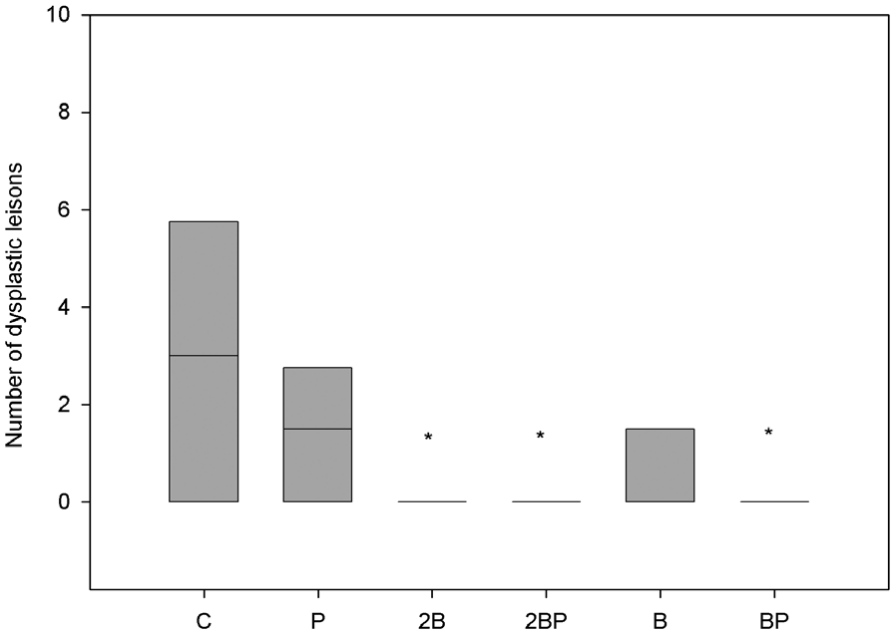
Quantification of dysplastic lesions. Number of dysplastic lesions in colon and rectum classified as low grade dysplasia in different treatment groups. * denotes P<0.05 compared to group C.

**Figure 6:**
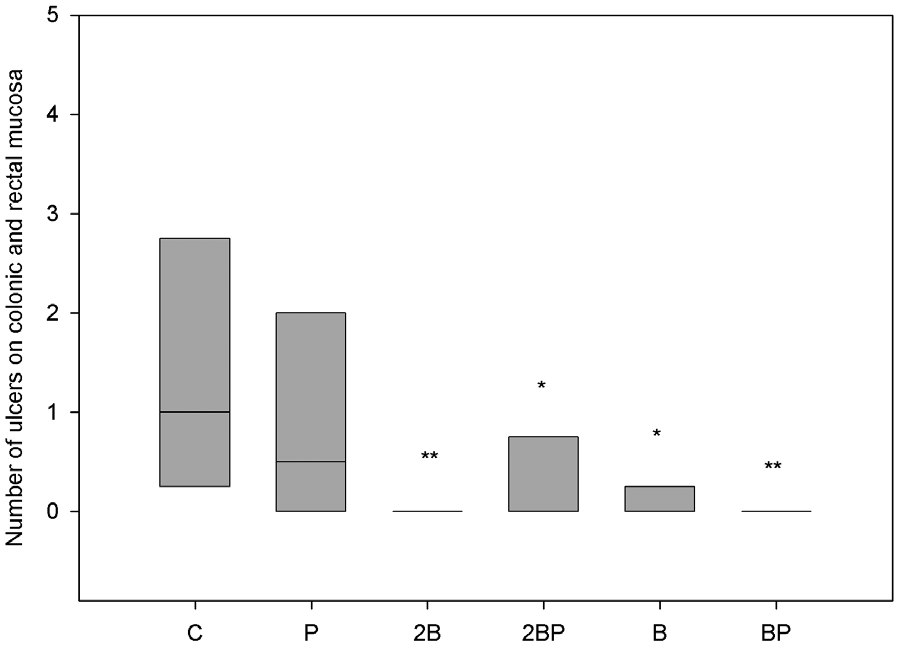
Quantification of colonic and rectal ulcers. Number of ulcers of colonic and rectal mucosa in different treatment groups. ** denotes P≤0.01 and * denotes P<0.05 compared to group C.

Quantitatively, the number of lesions classified as low-grade dysplasia was significantly reduced in groups 2B, 2BP and BP (P = 0.05) compared to group C (Fig. 5). A total of 26 dysplastic lesions distributed in 5 animals were found in group C, while the corresponding figures for group 2B were 2 lesions in one animal; for group 2BP and BP, 1 lesion for each group. A similar pattern was found for colonic ulcers, i.e. 11 ulcers were found distributed in 6 animals of group C, while no ulcers were found in group 2B group (P = 0.01); in group 2BP, 2 ulcers were found in 2 animals (P = 0.05); in group B, 1 ulcer was found (P = 0.043) and none in group BP (P = 0.01) (Fig. 6).

### Histopathological evaluation of the liver

**Figure 7:**
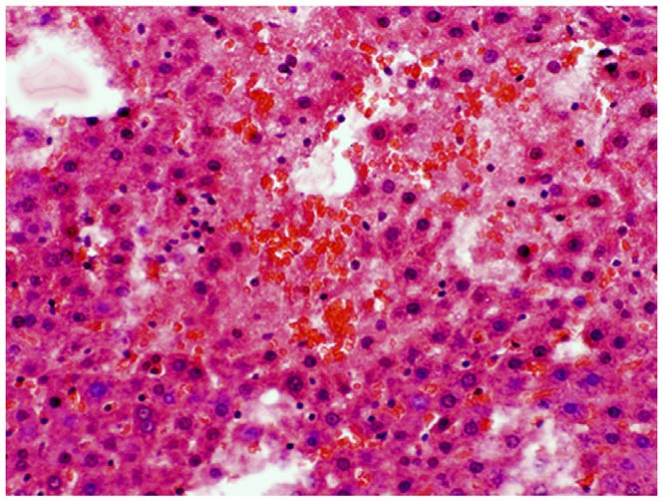
Liver injury group C. Focal parenchymal loss and haemorrhage in the liver of an animal in group C.

**Figure 8:**
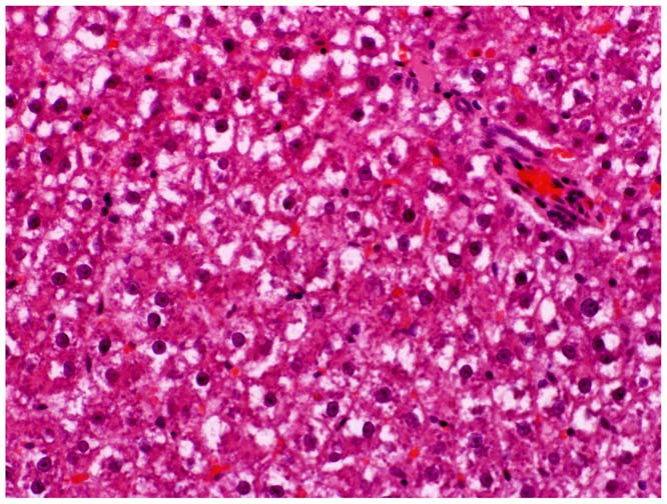
Liver injury group P. Hepatocytes showing displaced nucleus to the periphery, known as ballooning in an animal from group P.

**Figure 9:**
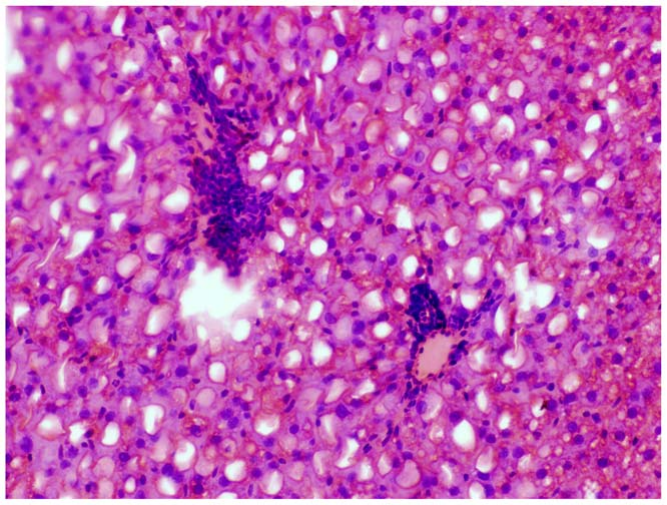
Liver injury group 2BP. Parenchymal inflammatory infiltration in steatotic areas from an animal in group 2BP.

Livers from group C showed mild to moderate degrees of steatosis. Liver lobules had occasional focal areas with parenchymal loss, haemorrhage, and small inflammatory infiltrations in non-steatotic areas (Fig. 7). Displaced nucleus to the periphery of the hepatocytes was occasionally found in livers from groups P (Fig. 8), B and BP. The overall histological changes in the different groups were similar to those of group C, but could be more or less severe. Based on scoring it was seen that, the degree of parenchymal inflammatory infiltration in non-steatotic area was significantly higher in group C (Fig. 7) compared to group 2BP (P = 0.038) (Fig. 9), group B (P = 0.019) and group BP (P<0.001) (Table 2). Significant increases in the degree of steatosis compared to group C was found in group P (P = 0.005) (Fig. 8) and in group BP (P = 0.004) (Table 2). Comparison of the total received score compared to maximum score revealed a significant increase of steatosis in groups P, 2BP and BP compared to rats fed corresponding diets without bacteria (P<0.01) (Table 2).

**Figure 10:**
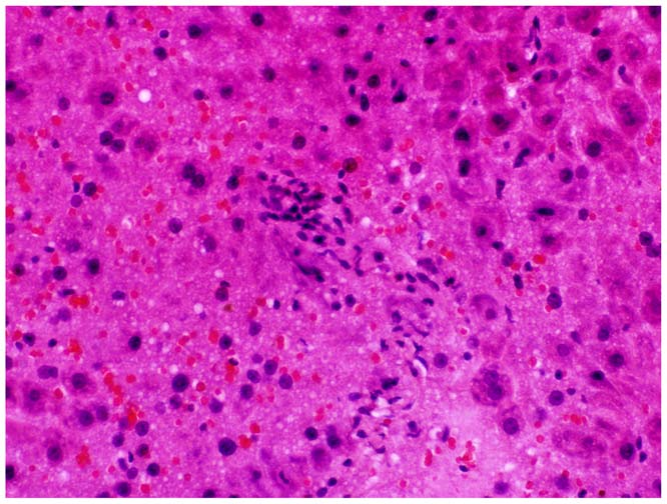
Liver injury group 2B. Parenchymal loss and haemorrhage in liver from an animal in group 2B.

The incidence of steatosis was in comparison with group C found to be significantly reduced in group 2B (P = 0.038) (Fig. 10, Table 3). Compared to group C, the incidence of stasis was decreased in group BP (P = 0.049) (Table 3), and so was also the incidence of translocation to the liver (P<0.05) (Table 4).

### Faecal viable count of *Enterobacteriacea* and lactobacilli

At the start (base line), the viable count of *Enterobacteriaceae* between groups did not show any significant differences. On the last day of the study the *Enterobacteriaceae* count was higher in group C (P<0.001) and in group B (P = 0.002), compared with their individual base line level, while this increase over time could not be seen in any of the other groups.

**Figure 11:**
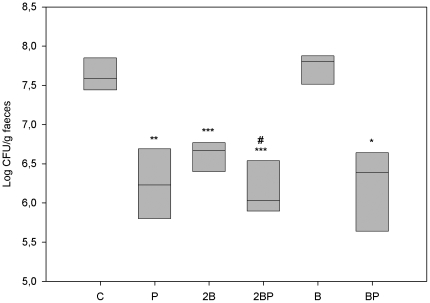
Faecal load of *Enterobacteriaceae*. Viable count of *Enterobacteriaceae* (log CFU/g faecal sample) *P<0.05, **P<0.01, *** P≤0.001 compared to group C. By the addition of probiotics: group C vs group P **P<0.01; group 2B vs group 2BP #P≤0.05; B group vs BP group *P<0.05.

At the end of the study, the count of *Enterobacteriaceae* was significantly decreased in groups P (P = 0.003), 2B (P<0.001), 2BP (P = 0.001) and BP (P = 0.017) compared with group C (Fig. 11). A significant decrease in *Enterobacteriaceae* count was achieved by the addition of probiotics to the diets, i.e. group C versus P (P = 0.003); 2B versus 2BP (P = 0.05); B versus BP (P = 0.036)) (Fig. 11).

**Figure 12:**
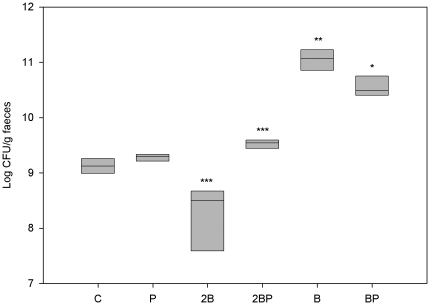
Faecal load of lactobacilli. Viable count of lactobacilli (log CFU/g faecal sample)*P<0.05, **P<0.01, ***P≤0.001 compared to group C. A decrease was found in group 2B *** P≤0.001 compared to group C. By the addition of probiotics: 2B group vs 2BP group ***P≤0.001, and B vs BP group *P<0.05.

At the base line, faecal viable count of lactobacilli differed between groups C and 2B (P = 0.003) and between groups 2B and 2BP (P<0.001). From the start to the end of the study, only group 2B exhibited a decrease in lactobacilli count (P<0.001), while it increased in groups B and BP (P = 0.002 and P = 0.012, respectively). At the last day of the study and compared with group C, the viable count was higher in groups 2BP (P = 0.001), B (P = 0.003) and BP (P = 0.017), and lower in group 2B (P = 0.001) (Fig. 12). The addition of probiotics to the 2B diet resulted in an increased viable count of faecal lactobacilli (P = 0.001) whereas it decreased when added to the B diet (P = 0.036) (Fig. 12).

### Identification of faecal lactobacilli

All identified isolates were designated to *Lactobacillus*. The different species found in the different animal groups at different time points are shown in Table 5. The probiotic supplement gradually changed the dominance of *L. murinus*. Isolates identified as *L. plantarum* and isolated from groups P, 2BP and BP were identified as the strain *L. plantarum* HEAL19 (Table 5).

**Table 5:** Identification to species level of faecal lactobacilli.

Group	Base line	After DSS cycle 1	After DSS cycle 10	Termination
C	*L. murinus*	*L. murinus*	*L. murinus*	*L. murinus*
	*L. murinus*	*L. murinus*	*L. murinus*	*L. murinus*
	*L. murinus*	*L. murinus*	*L. murinus*	*L. murinus*
	*L. murinus*		*L. murinus*	
P	*L. johnsonii*	*L. johnsonii*	*L. reuteri*	
	*L. johnsonii*	*L. plantarum*		
2B	*L. murinus*	*L. murinus*	*L. reuteri*	*L. murinus*
			*L. reuteri*	*L. murinus*
			*L. murinus*	*L. murinus*
				*L. murinus*
2BP	*L. gasseri*	*L. plantarum*	*L. plantarum*	*L. gasseri*
		*L. plantarum*	*L. plantarum*	*L. gasseri*
			*L. plantarum*	*L. plantarum*
B	*L. murinus*	*L. reuteri*	*L. murinus*	*L. johnsonii*
		*L. murinus*	*L. murinus*	*L. reuteri*
			*L. reuteri*	*L. johnsonii*
			*L. murinus*	
BP	*L. vaginalis*	*L. plantarum*	*L. murinus*	*L. plantarum*
	*L. vaginalis*		*L. plantarum*	
	*L. vaginalis*		*L. plantarum*	
			*L. plantarum*	

Identification of isolates from viable count of faeces from the different groups of rats.

(*L.* = *Lactobacillus*).

**Table 6:** SCFAs in colon content and blood samples.

	C	P	2B	2BP	B	BP
Caecum						
Acetic	66.0^a^	64.9	75.6^b^	75.2	69.0^a^	69.6
Propionic	16.9^a^	16.1	15.1^a,b^	14.8	13.5^b^	12.9
Butyric	17.1^a^	19.1	9.2^b^	9.9	17.6^a^	17.5
Total (µmol/g)	82.0^a^	89.7	55.3^b^	59.8	75.3^a^	80.4
Proximal colon						
Acetic	69.5^a^	70.9	76.0^b^	77.8	70.9^a^	72.7
Propionic	16.1	13.6	15.2	13.5	13.4	11.8
Butyric	14.4^a^	15.5	8.8^b^	8.8	15.7^a^	15.5
Total (µmol/g)	74.3^a^	73.9	56.0^b^	63.7	72.8^a,b^	75.3
Distal colon						
Acetic	66.4^a^	66.0	73.1^b^	73.0	67.5^a^	69.5
Propionic	18.4	19.1	16.6	15.6	14.5	14.5
Butyric	15.3^a^	14.9	10.3^b^	11.5	18.0^a^	16.0
Total (µmol/g)	75.0^a^	75.8	38.6^b^	47.6	82.1^a^	70.5
Blood from aorta^1^						
Acetic	97.0	97.5	97.9	97.4^*^	97.9	98.0
Propionic	1.1	0.7^***^	0.7	0.8^**^	0.7	0.6
Butyric	1.9	1.8	1.5	1.8	1.4	1.4

Proportions (%) of acetic-, propionic- and butyric acids in the hindgut and blood of rats given diets supplemented with different amounts of blueberry husks (2B = 122 g blueberry/kg dwb and B = 61 g blueberry/kg dwb) and/or probiotics (P; 6⋅10^9^ CFU per day).

Probiotics: *Bifidobacterium infantis* (CURE21), *Lactobacillus gasseri* VPG44, and *Lactobacillus plantarum* (HEAL19).

Mean values for eight rats per group, B group (n = 6).

Mean values of C, 2B and B only, i.e. those without any added probiotics, with unlike superscript letters were significantly different (P<0.05).

Mean values were significantly different from those rats fed diets without bacteria: *P<0.05, ** P<0.01, *** P<0.001.

1 2B (n = 7), B and BP (n = 6).

### Proportions of SCFAs in the hindgut and in blood

The proportions of acetic acid, propionic acid and butyric acid were in average 65%, 14% and 14% in caecum, 70%, 13% and 12% in proximal colon, 66%, 16% and 13% in distal colon, and 95%, 1% and 2% in aortic blood (Table 6).

Generally, higher proportions of acetic acid (P<0.05) and lower proportions of butyric acid (P<0.05) was found in the hindgut of group 2B compared with groups C and B (Table 6). The proportions of propionic acid in aortic blood was significantly decreased in the P group compared with the C group (P<0.001) and in the 2BP group compared with the 2B group (P<0.01) (Table 6).

### Levels of SCFAs in the hindgut

In general total levels of SCFAs along the hindgut were lower in rats fed 2B than in rats fed C (P<0.05), due to a lower content of most individual acids. Compared with B the butyric acid levels were lower in the caecum of rats fed 2B (P<0.001) and in distal part of colon this was valid for both propionic and butyric acid (6.1±0.6 vs 11.3±3.2 µmol/g and 3.7±0.3 vs 15±5.7 µmol/g; P = 0.045 and P<0.001, respectively). The butyric acid level in the distal part of colon was higher in rats fed 2BP than in rats fed only 2B (5.0±0.3 vs.3.7±0.3 µmol/g; P = 0.005).

### Levels of SCFAs in aortic blood

Acetic acid was the major acid in aortic blood in all groups (in mean 1152.6 µmol/l) followed by butyric acid (in mean 18.7 µmol/l) and propionic acid (in mean 8.8 µmol/l).

The level of propionic acid was higher in group C than in groups 2B and B (P<0.05). The propionic acid levels were significantly lower in group P (8.0±0.4 µmol/l) compared with group C (11.2±0.2 µmol/l) and in group 2BP (8.3±0.3 µmol/l) compared with group 2B (9.5±0.4 µmol/l) (P<0.001 and P<0.05, respectively).

## Discussion

It has previously been demonstrated that dysplasia and adenocarcinomas can be induced by cyclic administration of DSS [19]. DSS is not mutagenic [33], and the changes occurring depend on the mucosal inflammatory ulceration and regeneration, recurrence-remission cycles typical of clinical ulcerative colitis cases [34]. Developing therapeutic regimens to combat colorectal cancer without significant side effects is of great interest, and in the current study we used a similar model to determine the protective effect of blueberry husks with and without addition of a probiotic mixture to delay or prevent colon carcinogenesis, and pathological abnormalities of the liver.

The clinical findings during the first cycle of DSS administration showed lower scoring values for groups 2B, 2BP and B compared with the C group (Fig. 1). A gradually increased inflammatory activity was recorded in all groups, indicated by a significant increase in the DAI from cycle 1 through 11 (Fig. 1). There is a well-characterised sequence of changes in the DSS model, beginning with increased mucosal permeability, and eventual ulceration followed by inflammation. After DSS withdrawal, these events are followed by epithelial restitution, cell migration and proliferation. The supposed impaired recovery periods between exposures to DSS may suggest a chronological sequence, where repeated uninhibited acute inflammatory responses with less extension of mucosal repair develop chronicity. At the last DSS cycle, the DAI were significantly lower in groups administered supplementary blueberry husks with or without probiotics (Fig. 1).

High level of haptoglobin has been implicated in patients with colorectal cancer and development of hepatic metastases and it has been stated to be a sensitive indicator [35]. In the present study, a successive rise in the concentration of haptoglobin in serum was observed for all groups, from the beginning to the end of the study period. During the 10^th^ cycle of DSS administration, group 2B showed significantly lower values than group C, but also group 2BP had a reduced level that was close to significance (P = 0.054) (Fig. 2).

The pathogenesis of colorectal carcinogenesis associated with colonic inflammation is believed to involve progression from inflamed and hyperplastic cryptal cells, through dysplasia, to adenoma and carcinoma [36]. Strong circumstantial evidence documents the validity of UC-associated dysplasia as a precursor lesion or marker of carcinomas, and it is likely that colorectal carcinomas evolve through stages of increasingly severe epithelial dysplasia before becoming invasive lesions [37]. According to the present criteria, microscopic findings of descending colon revealed histological abnormalities with low and high grade dysplasia and adenocarcinomatous polyps in groups C, P, B and BP (Fig. 3, 4), whereas only sections with low-grade dysplasia were found in groups 2B and 2BP. Macroscopic evaluation showed a significantly lower number of low-grade dysplastic lesions compared to group C in colon of groups 2B, 2BP and BP (Fig. 5), as well as fewer mucosal ulcers in groups 2B, 2BP, B and BP (Fig. 6).

Increased gut permeability can be explained by malignant tissue disruption of the bowel architecture [38], making gut-derived bacteria and toxins accessible to the liver via the portal circulation [39]. According to the present liver scoring, the degree of parenchymal infiltration was significantly higher in group C compared to groups 2BP (Fig. 7, 9), B, and BP (Table 2), and the incidence of translocation to the liver was lower in group BP (Table 4). In fact, no translocated bacteria were found in this group. Among the identified bacteria isolated from the liver, both *Enterococcus* and *Clostridium* spp. were found. *Enterococcus* spp. are important causes of human infections [40] and different species of *Clostridium* have been implicated in the induction of intestinal inflammation, due to production of extracellular toxins, and they may be detrimental during increased permeability of the colonic mucosa [41]. It should be pointed out, that group 2BP was the only group where no *Clostridium* was found in the liver (Table 4). A significantly reduced degree of parenchymal cellular infiltration was also found in this group (Table 2). These findings may be significant because *Clostridium* bacteriemia have been evident in many patients with a diagnosis of gastrointestinal or hematologic malignancy [42]. A gastrointestinal source of infection, particularly carcinoma of the colon or rectum or enterocolitis, was evident in most patients [42]. Fatty infiltration of hepatocytes has been reported in intestinal inflammation [6]. The incidence of steatosis in the present study was found to be significantly reduced in group 2B (Fig. 10, Table 3), compared to group C (Fig. 7), while the scoring of steatosis was increased in groups P (Fig. 8) and BP (Table 2).

Okayasu *et al*., [34] presented significant increases in *Enterobacteriaceae* and *Clostridium* spp. in faeces during DSS administration and *C. ramosum* were particularly evident after repeated administrations. Also, *C. ramosum* has been one of the most frequently isolated anaerobes from the inflamed mucosa of UC patients, against which an enhanced antibody response was found [43]. In a study by Sokol *et al.*, [44], the biodiversity of the active microbiota was shown to be lower for UC patients than for healthy controls and *Escherichia coli* (or related *Enterobacteriaceae*) were significantly associated with UC. In the present study, the viable count of *Enterobacteriaceae* in faeces increased during the experimental period in group C but also in group B. However, at the end of the study, a significant decrease was obvious in all groups, except for group B, compared to group C (Fig. 11). Also, the addition of probiotics seems to suppress *Enterobacteriacea* (Fig. 11). The action of LPS from gram-negative bacteria is effectuated via the toll-like receptor 4 (TLR4) protein and a decrease in the load of *Enterobacteriaceae* must be regarded as an advantage, since it has been demonstrated that TLR4 expression is up-regulated in colitis-associated cancer lesions from patients with UC [45]. In active UC patients, the numbers of faecal lactobacilli decrease, indicating that a reduction in intestinal *Lactobacillus* species may be a sign of mucosal inflammation [9]. In the present study, the viable count of faecal lactobacilli at the end of the study period and in comparison with group C, was higher in groups 2BP, B and BP, but lower in group 2B (Fig. 12). The addition of probiotics increased the viable count of lactobacilli in group 2BP compared to group 2B (Fig. 12). In contrast, a decrease was found between groups B and BP (Fig. 12). Changes in luminal concentrations of phenolics can affect the microbiota composition [17] and the growth of certain bacterial groups with pathogenic potential. For example, *Enterobacteriaceae*, *Clostridium* and *Bacteroides* can be repressed by phenolics, while some probiotics are relatively unaffected. This may favour intestinal establishment of some probiotics, inhibition of pathogens and improvement of the bacterial balance of the gut [17]. An increase in numbers of *E. coli* and clostridia in patients with active UC, may contribute to pathogenesis [8]. In the present study, the added strain of *L. plantarum* was isolated and identified from faeces of animals that had been supplemented with probiotics (Table 5). Since *Clostridium* was not found in the livers of group 2BP (Table 4), it can be hypothesised that *L. plantarum* had a beneficial effect that supplemented that of the blueberry husks. The results of Russel *et al*., [46] suggest that the protective anti-inflammatory effect of blueberry associated phenolics in colon, probably occur due to microbial metabolism, which in turn is dependent on the individual composition of the microbiota.

The formation of SCFAs is mostly regulated by substrate availability and composition of the microbiota. Lower proportions of butyrate and higher proportions of acetate have been found in patients with adenomatous polyps [47], suggesting an increased floral capacity to produce acetate and a decreased one in the capacity to form butyrate [47]. In fact, the capacity of stool-derived bacteria from patients with colonic adenomas and colon cancer to produce butyrate was significantly reduced [48]. In the present study, the proportion of butyric acid was decreased and that of acetic acid increased in caecum, proximal and distal colon of group 2B compared with groups C and B (Table 6), and the same trend was seen in the 2BP group (Table 6). The utilisation of butyrate, the major energy substrate for colonocytes, is significantly impaired during both severe colitis [49] and during hepatic cirrhosis [50]. Negative correlations between lipid peroxidation and butyric acid as well as between DAI and butyric acid have also been shown in DSS-induced acute colitis [24]. However, histological examination revealed less epithelial affection in groups 2B and 2BP compared with the other groups, which may explain the superior disposal of butyrate in these groups, as judged by the findings of lower proportions of butyric acid. The high content of uronic acids in blueberry husks could explain the increased proportion of acetic acid (Table 6).

The increased concentration of propionic acid in serum of patients with chronic hepatitis of various etiology at the stage of hepatic cirrhosis with hepatic encephalopathy syndrome, has been shown to correlate with the results of clinical and laboratory methods [51]. In the present study, the addition of probiotics to the diet, significantly decreased the level of propionic acid in aortic blood in group P and group 2BP, compared with groups C and 2B, respectively. When the proportion of propionic acid in aortic blood was taken into account, lower values were found in all treatment groups (Table 6).

Fat accumulation in the liver of hypercholesterolaemic rats fed oat fibres has been shown earlier [52] and the accumulation was observed along with an increase in the concentration of oat. This might be caused by the increased fatty acid and cholesterol synthesis, which is enhanced in the liver when feeding soluble oat fibre [53]. The increase in fatty acid synthesis was due to the increased flow of SCFAs in the liver and a positive correlation between plasma propionate concentrations and hepatic fatty acid synthesis was found [53]. However, no cirrhotic, necrotic or inflammatory changes were observed so, in this model, the livers were not otherwise affected [53]. In the present study, the liver was highly affected from chronic intestinal inflammation, which probably impairs normal function. The addition of probiotic bacteria caused a significant increase in total scoring of steatosis compared to those animals fed diets without bacteria (Table 2), but the concentration of propionic acid in the blood decreased. It can be speculated that the livers from groups P, 2BP, B and BP have better preserved capability of fatty acid synthesis, which may reflect an improved liver capacity.

In conclusion, DAI was significantly decreased by blueberry husks administered solely or along with probiotics. The use of blueberry husks alone or in association with probiotics reduced the number of dysplastic lesions and mucosal ulcers. The probiotic mixture could decrease the load of faecal *Enterobacteriaceae* and increase that of lactobacilli. The colonic epithelium of groups given the high dose of blueberry, with or without probiotics, was less affected. In the same groups, haptoglobin levels were decreased. Furthermore, the probiotic mixture seems to provide protection against hepatic damage. Our data indicates a therapeutic option for use of blueberry husks and probiotics to delay colonic carcinogenesis and the subsequent hepatic damage, at least in the applied animal model.
